# Supplementary material for: Translocation mechanism of xeroderma pigmentosum group D protein on single-stranded DNA and genetic disease etiology
Source: Nat Commun. 2025 Nov 28;16:11703. doi: 10.1038/s41467-025-66834-1 (PMC12753804; doi:10.1038/s41467-025-66834-1)
Supplement: Supplementary file 1 — Supplementary Information [file 41467_2025_66834_MOESM1_ESM.pdf]

SUPPLEMENTARY INFORMATION

Molecular mechanism of xeroderma pigmentosum group D protein translocating on single-stranded DNA and implications for genetic diseases

Tanmoy Paul[1,2], Chunli Yan[1,2], Grant Derdeyn-Blackwell[1,2], and Ivaylo Ivanov[1,2*]

[1] Department of Chemistry, Georgia State University, Atlanta, Georgia, USA.

[2] Center for Diagnostics and Therapeutics, Georgia State University, Atlanta, Georgia, USA.

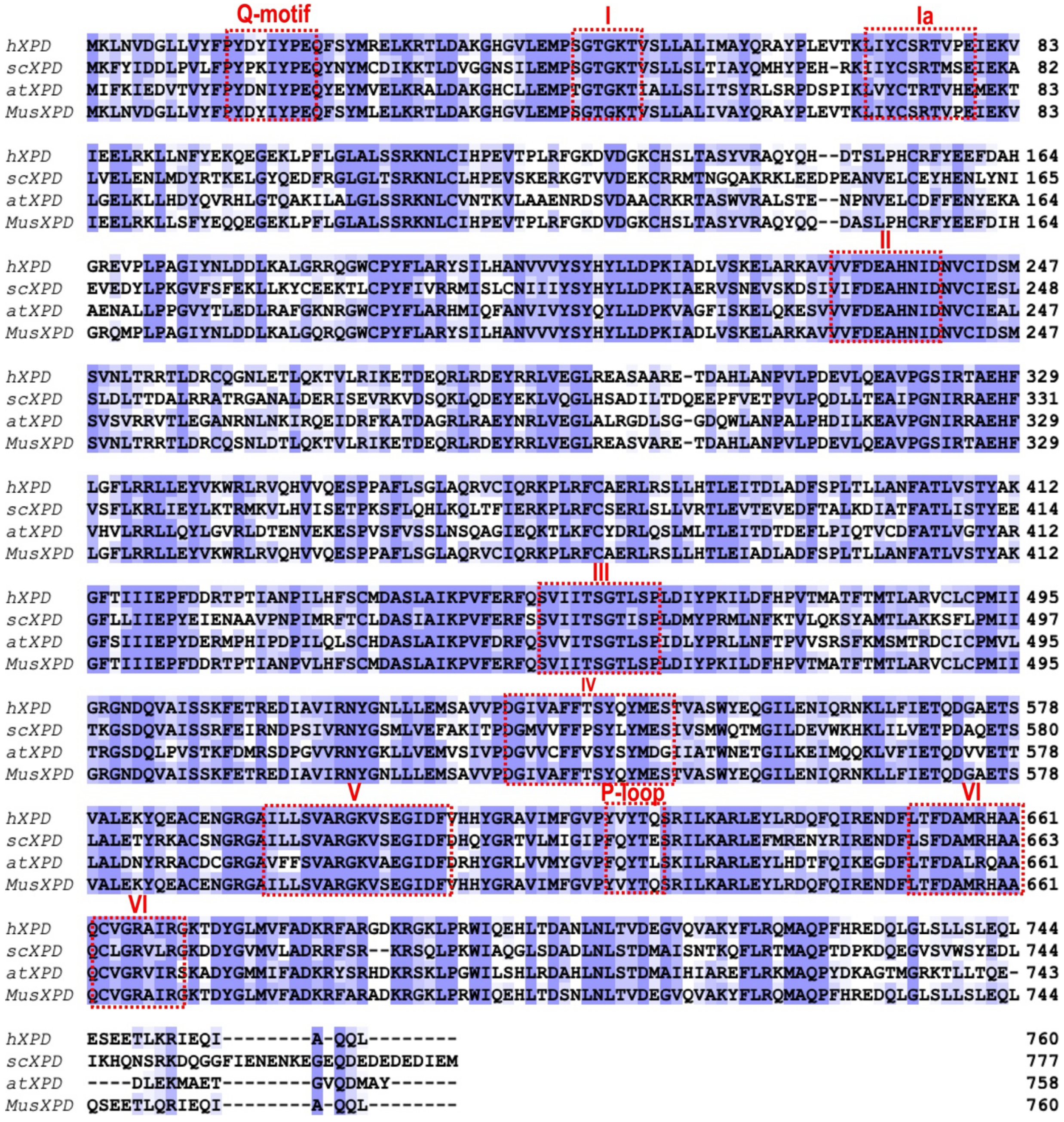

Supplementary Figure 1. Sequence alignment of XPD with SF2 family helicases. Canonical conserved helicase motifs are highlighted by red dashed line boxes. Abbreviations: hXPD, human XPD; scXPD, *Saccharomyces cerevisiae* XPD; atXPD, *Arabidopsis thaliana* XPD; MusXPD, *Mus musculus* XPD.

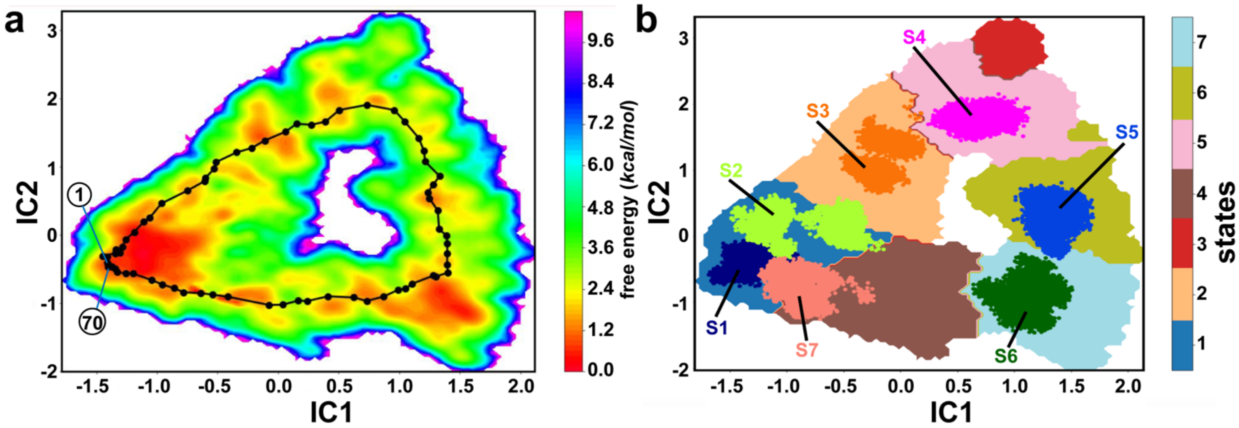

Supplementary Figure 2. Free energy landscape reflecting the protein conformational dynamics during XPD's ATPase cycle. a Free energy landscape projected onto the first two independent components (ICs) obtained from time-lagged independent component analysis. TICA analysis was based on distances from the center of mass of the RecA1 domain to all Cα atoms of the RecA2, Arch, and Fe–S domains. The color bar indicates the free energy scale in units of kcal/mol. **b** Markov state model (MSM) derived from the XPD conformational ensemble sampled along the optimal transition path. Macrostates representing the functional states of XPD were obtained with the PCCA+ algorithm and color-coded as indicated on the color bar. Microstates (dots) from Figure 2 were mapped onto the two ICs defined over the protein conformational space. This mapping establishes correspondence with the MSM analysis performed in the complete space defined by the protein–nucleic acids distances.

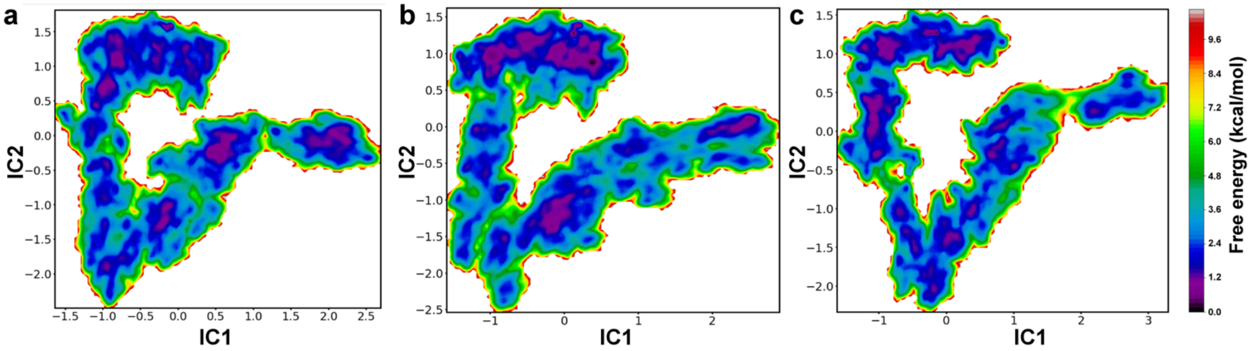

Supplementary Figure 3. Three independently sampled XPD ATPase cycles show practically identical dynamics. Free energy profiles corresponding to three independent XPD ATPase cycles: (**a**) cycle 1; (**b**) cycle 2; (**c**) cycle 3. Each cycle is projected onto a subspace defined by the first two ICs computed over all distance from protein Cα atoms to nucleic acid P atoms. Each cycle successively shifts the ssDNA by one nucleotide along XPD's DNA binding groove. The color bar shows the free energy scale in units of kcal/mol.

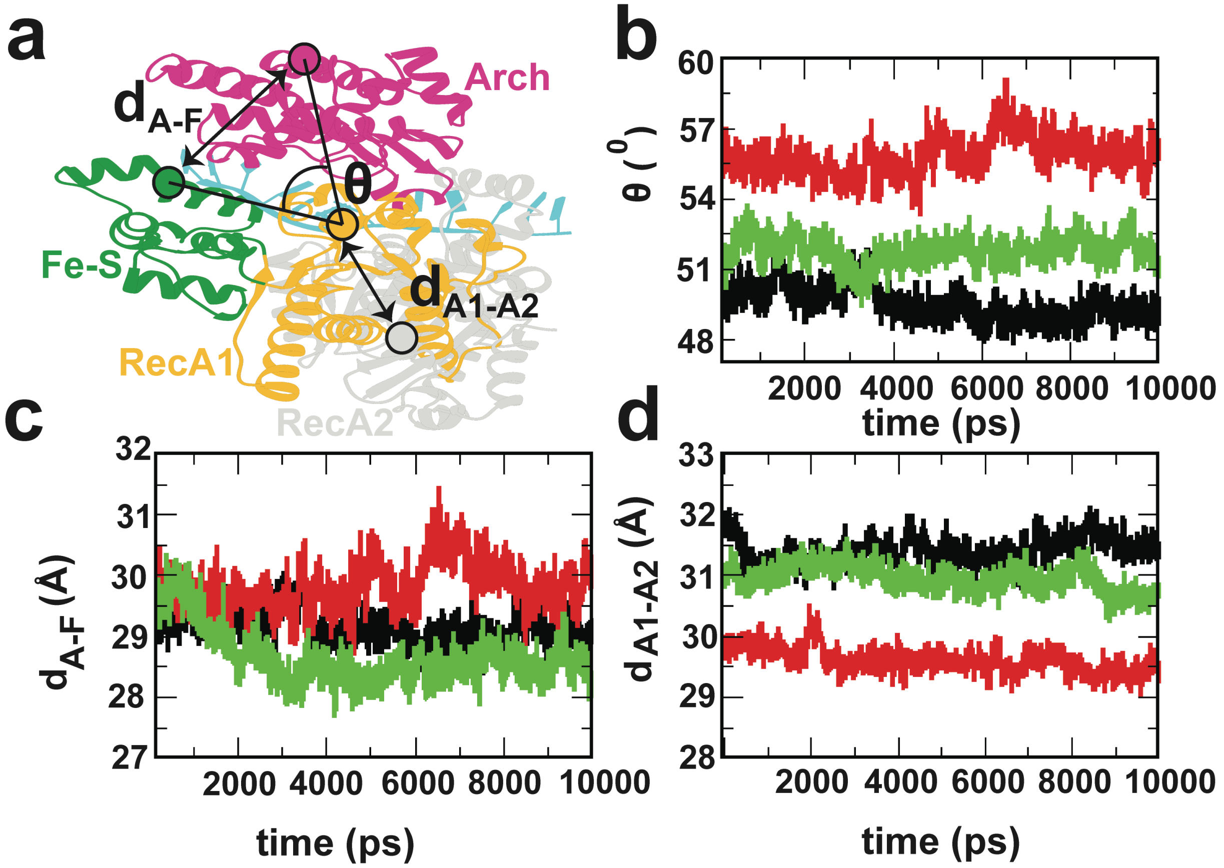

Supplementary Figure 4. Changes in characteristic geometric parameters reveal the large-scale domain motions of XPD during the ATPase cycle. a Schematic defining the geometric parameters that were used to monitor XPD domain rearrangements. These include The COM angle (θ) defined by the Arch, Fe–S and RecA1 domains and the COM distances between the Arch and Fe–S ($d_{A–F}$) domains and the two RecA domains ($d_{A1–A2}$), respectively. The centers of mass (COM) of XPD's domains are indicated with black circles. **b** The θ angle plotted as a function of MD trajectory frame. **c** The $d_{A–F}$ distance plotted as a function of MD trajectory frame. **d** The $d_{A1–A2}$ distance plotted as a function of MD trajectory frame. The apo state (S1) is shown by a black line, the ADP-bound state (S5) by the green line, and the ATP-bound state (S4) by the red line. Source data are provided as a Source Data file.

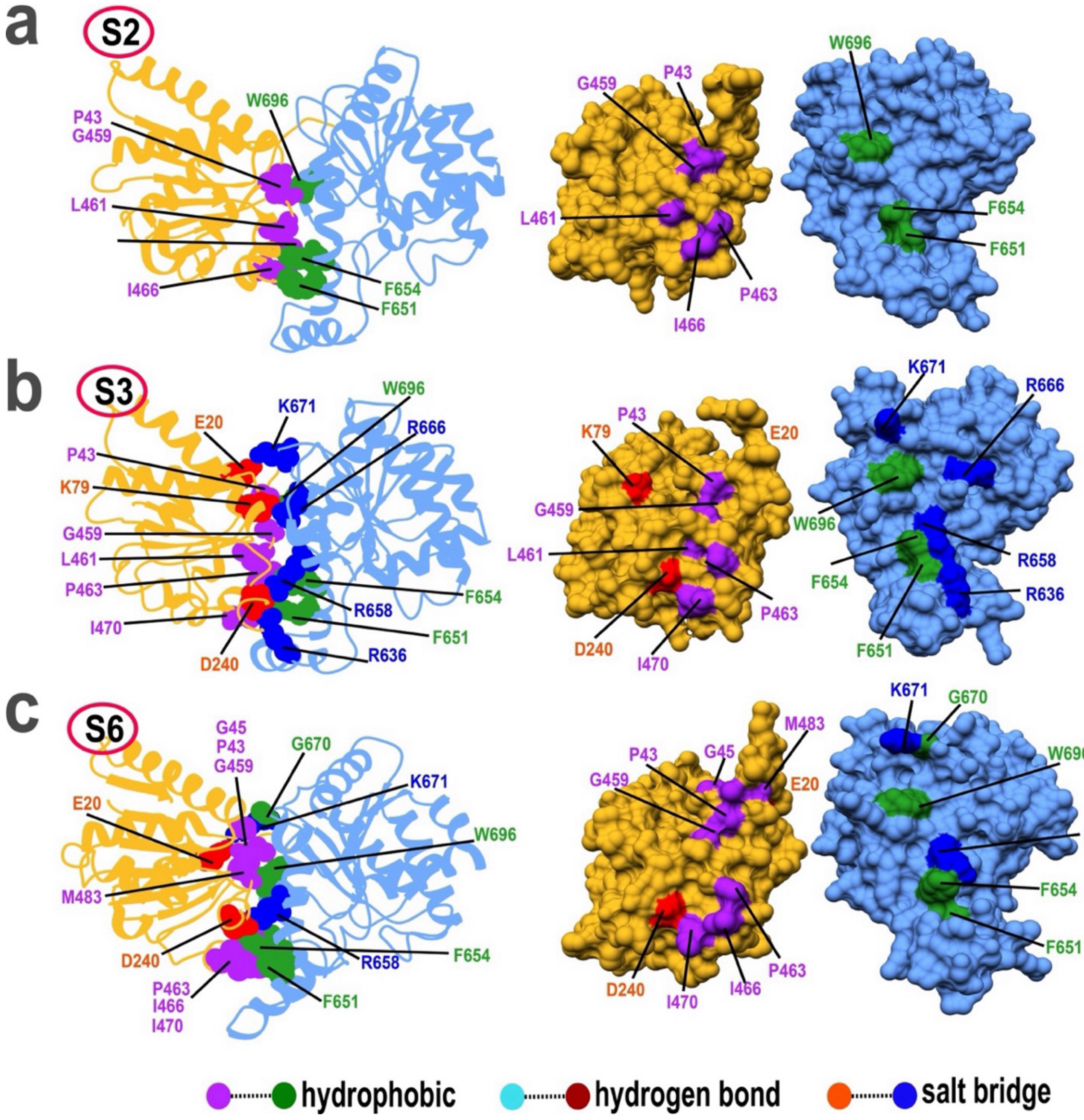

Supplementary Figure 5. Persistent residue contacts at the RecA1–RecA2 interface for multiple functional states. Persistent contacts at the XPD motor domain interface for MSM macrostates: (**a**) S2, (**b**) S3, and (**c**) S6. Panel on the left show key interacting residues explicitly in atomic representation (as spheres). Panels on the right show these residues mapped onto the domains' molecular surfaces in an 'open-book' view. Contacts are color-coded by type of interaction: hydrophobic interactions (purple–green), hydrogen bonding interactions (cyan–dark red), and salt bridge interactions (red–blue). RecA1 and RecA2 domains are depicted in gold and blue, respectively.

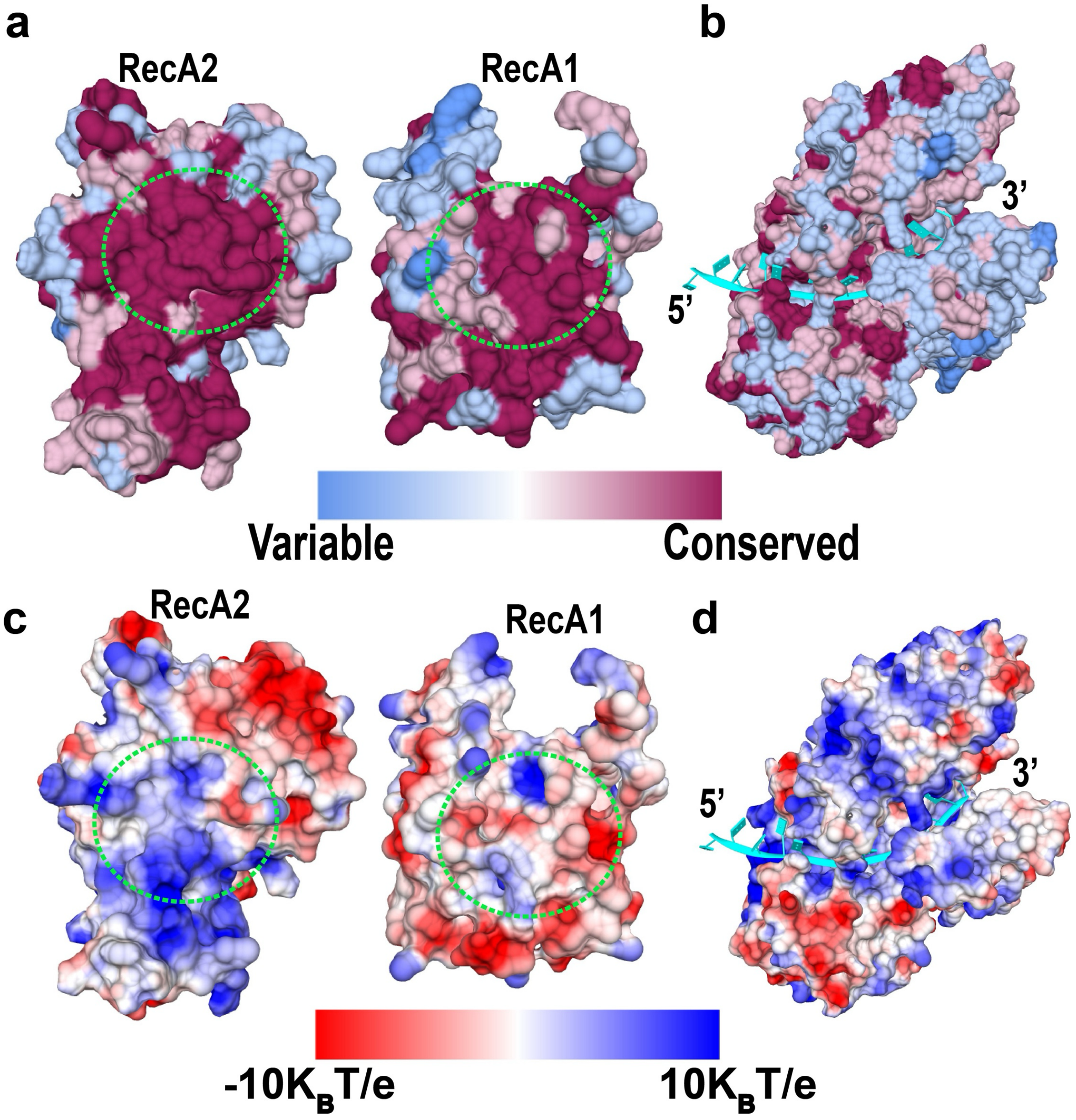

Supplementary Figure 6. Conservation and electrostatics maps of the motor domain interface and ssDNA binding groove of XPD. Conservation map of (**a**) the RecA1-RecA2 interface and (**b**) the ssDNA binding groove of XPD. Electrostatic potential mapped onto (**c**) the RecA1-RecA2 interface, and (**d**) the ssDNA binding groove of XPD. Conservation maps were generated by aligning the sequences from Supplementary Figure 1. Electrostatic potentials are mapped onto the molecular surface with negative values in red and positive values in blue.

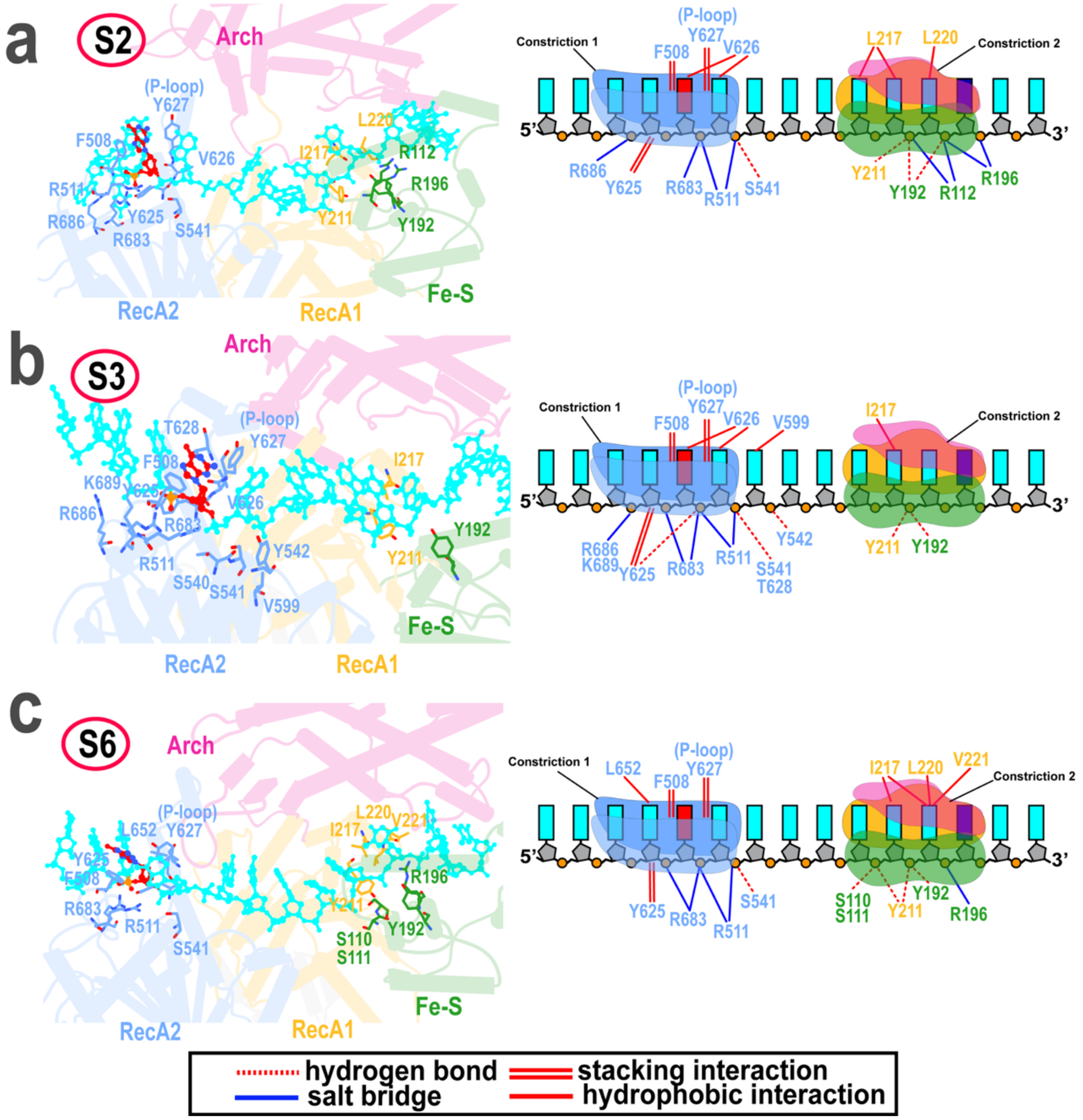

Supplementary Figure 7. XPD–ssDNA interactions across for multiple functional states. Persistent contacts between XPD and ssDNA are shown for macrostates (**a**) S2, (**b**) S3, and (**c**) S6. Panels on the left show the interacting XPD residues in atomic (stick) representation colored by domain. XPD domains are colored as follows: RecA1 (gold), RecA2 (blue), Arch (magenta), and Fe–S (green). Panels on the right show the XPD–ssDNA interactions as a schematic with contacts classified as hydrogen bonds (red dashed lines), salt bridges (blue solid lines), stacking interactions (red parallel lines), and hydrophobic contacts (red solid lines). Constriction 1 (proximal to the 5′-end of ssDNA) and Constriction 2 (near the 3′-end of ssDNA) are shown as outlines and colored according to the contributing domains.

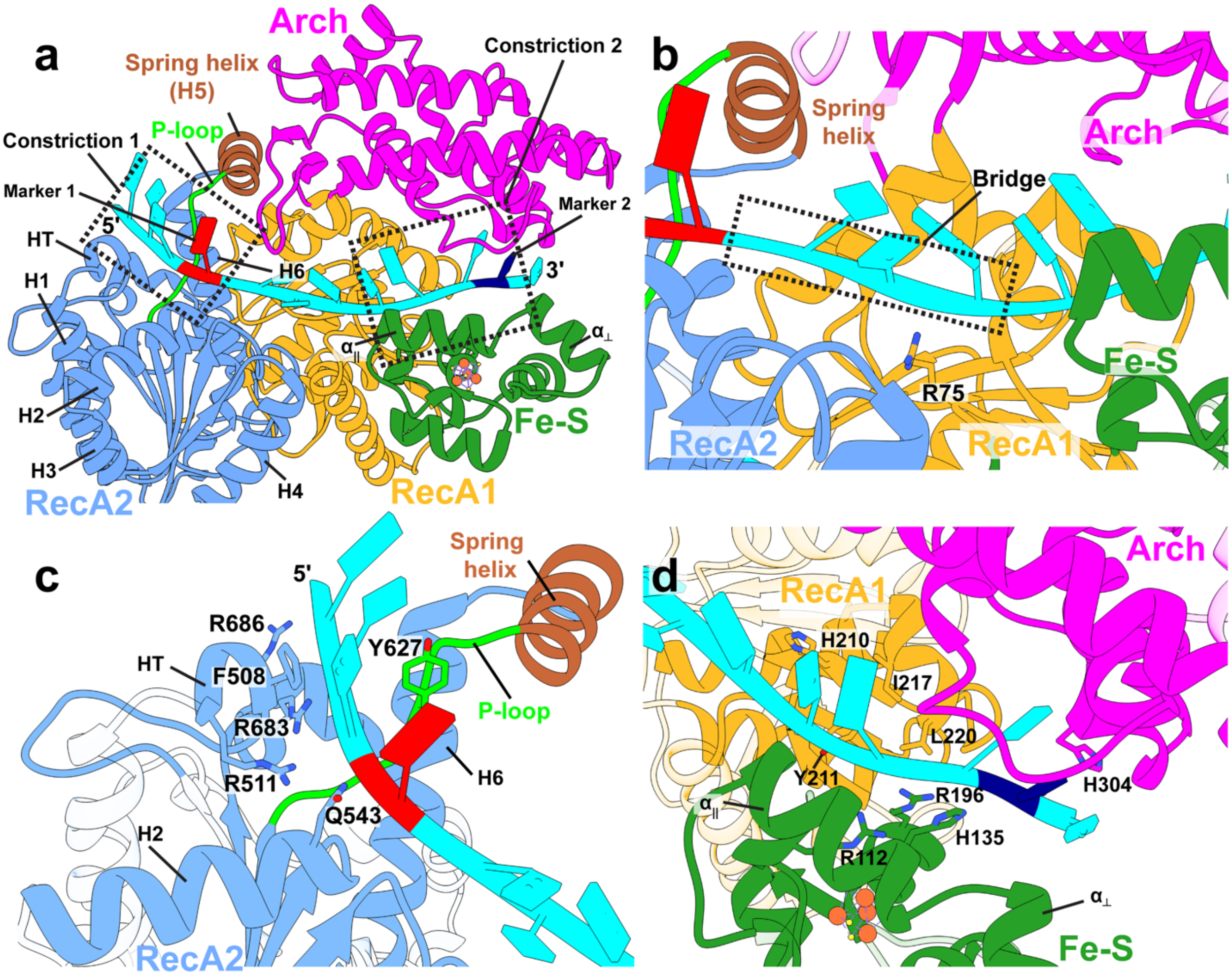

Supplementary Figure 8. Coordination controlling structural elements of XPD that enable ordered translocation on ssDNA. a Constriction 1 and Constriction 2 are essential for XPD translocation and are shown by black dashed rectangles superimposed on the XPD structure. XPD domains are colored as follows: RecA1 (gold), RecA2 (blue), Arch (magenta), and Fe–S (green). Helices within RecA2 and the HT helical turn are labeled. The spring helix is shown in brown; the P-loop is shown in light green. The first nucleotide entering each constriction is labeled and color-coded: Marker 1 (red) for Constriction 1 and Marker 2 (navy blue) for Constriction 2. **b** Zoomed-in view of the bridging ssDNA segment between the two constrictions. The R75 residue forming a persistent contact with the bridge is labelled. **c** Zoomed-in view of Constriction 1, showing the key secondary structure elements and amino acid residues interacting with ssDNA. **d** Zoomed-in view of Constriction 2 showing the key secondary structure elements and amino acid residues responsible for ssDNA binding.

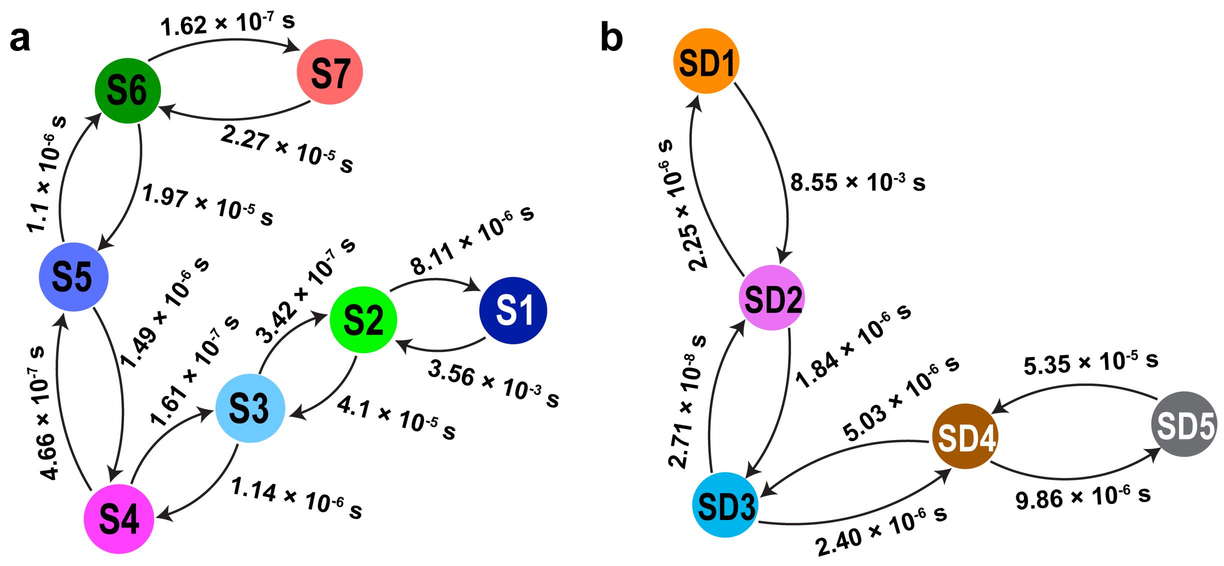

Supplementary Figure 9. Forward and backward transition timescales between macrostates from mean first passage time analysis for a) XPD and b) DinG. Vertices denote MSM macrostates. Directed edges (black arrows) represent forward and backward transitions between pairs of macrostates. Edges are labelled with the corresponding timescale values.

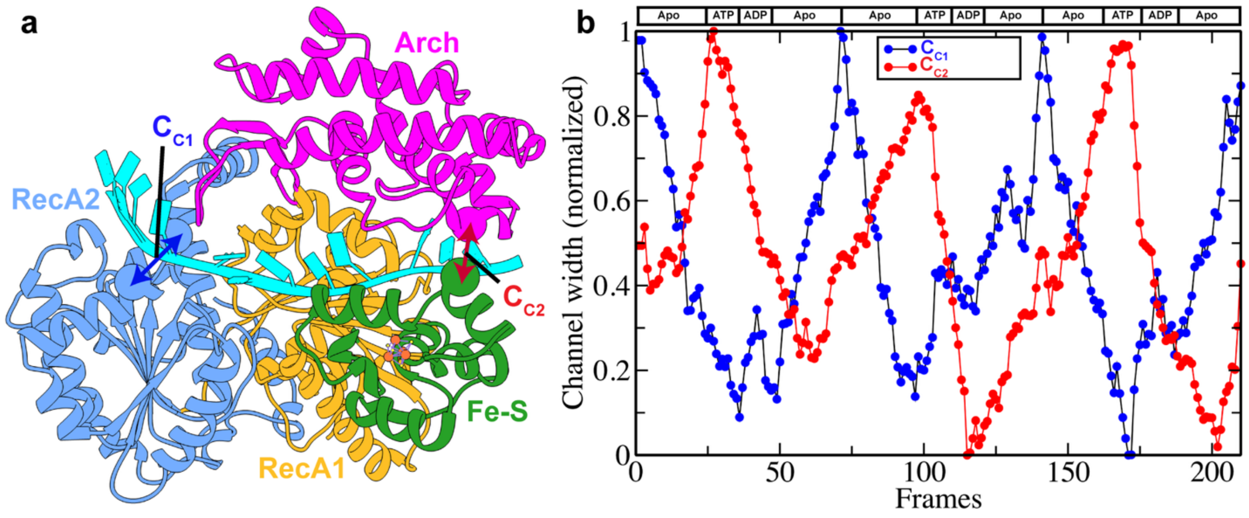

Supplementary Figure 10. Alternating binding affinities at Constriction 1 and Constriction 2 during XPD's three consecutive ATPase cycles. Channel widths at Constriction 1 and Constriction 2 (denoted as C_{C1} and C_{C2} respectively) are plotted as a function of trajectory frame for three consecutive ATP binding and hydrolysis cycles, each advancing ssDNA by one nucleotide. Channel widths are defined as follows: C_{C1} is measured as the shortest Cα–Cα distance between the H2 and H6 helices of the RecA2 domain in the apo state. C_{C2} is measured as the shortest Cα–Cα distance between the $\alpha_{\perp}$ helix of the Fe–S domain and a short helix within the Arch domain (residues 300–305) in the apo state. **a** The locations of the C_{C1} and C_{C2} distances superimposed on the XPD structure colored by domain. **b** Normalized channel width fluctuations at C_{C1} (blue) and C_{C2} (red). Distinct nucleotide states of XPD are annotated at the top of the graph. Source data are provided as a Source Data file.

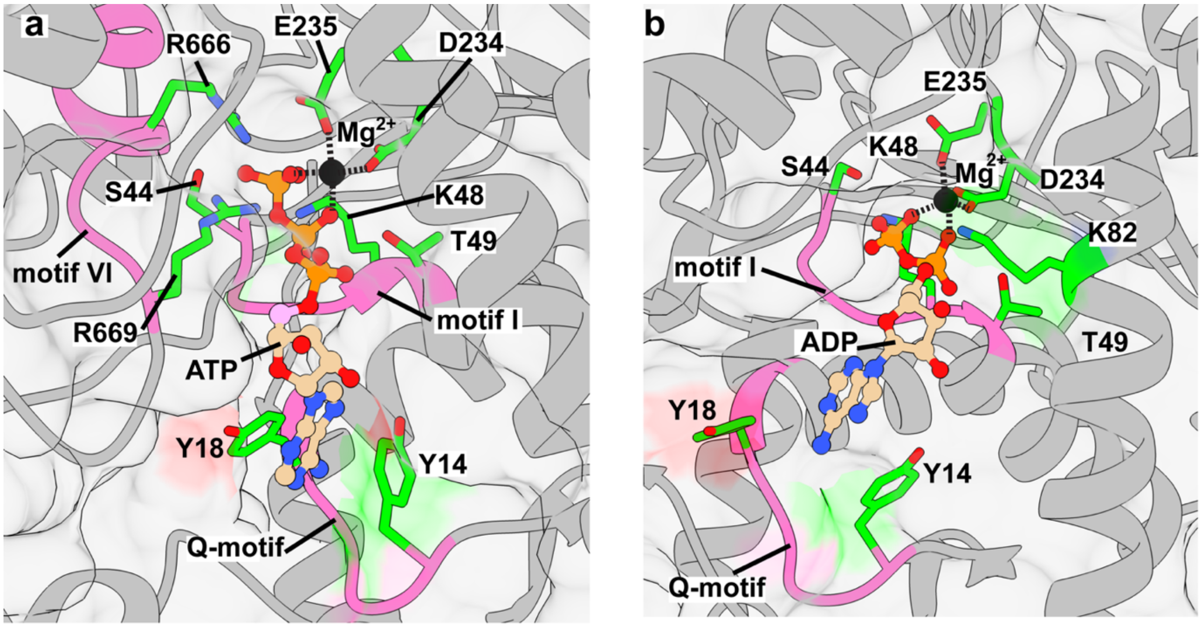

Supplementary Figure 11. Persistent residue contacts to the bound nucleotide in the XPD ATPase site. The XPD active site shown with bound nucleotide: (**a**) ATP and (**b**) ADP, respectively. The nucleotide is shown in ball-and-stick representation. Interacting residues are shown in stick representation and colored in green. The conserved helicase motifs involved in ATP and ADP binding are shown in pink and labeled.

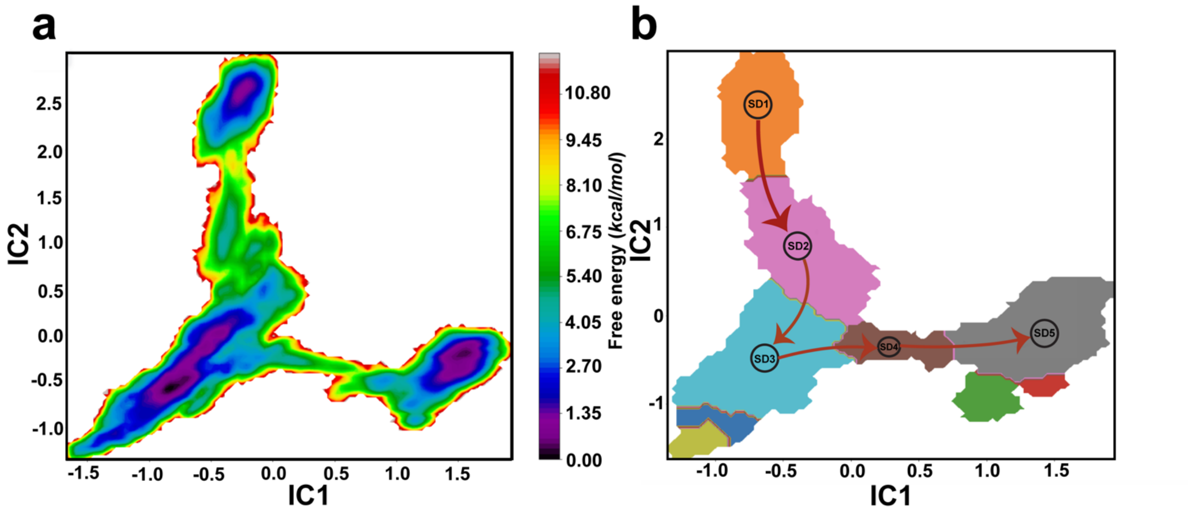

Supplementary Figure 12. Five on-path conformational states define the DNA translocation mechanism of DinG. a Free energy landscape underpinning DinG dynamics over a single ATPase cycle. The free energy is projected onto the first two ICs obtained from time-lagged independent component analysis. The color bar represents the free energy scale in kcal/mol. **b** Markov state model (MSM) built from the conformational ensemble sampled along the optimal transition path. Macrostates are assigned using the PCCA+ algorithm and colored. Five macrostates (SD1-SD5) traversed by the minimum free energy path (MFEP) are labeled. The remaining macrostates (in dark blue, green, red and olive yellow) correspond to kinetic traps and are not labeled.

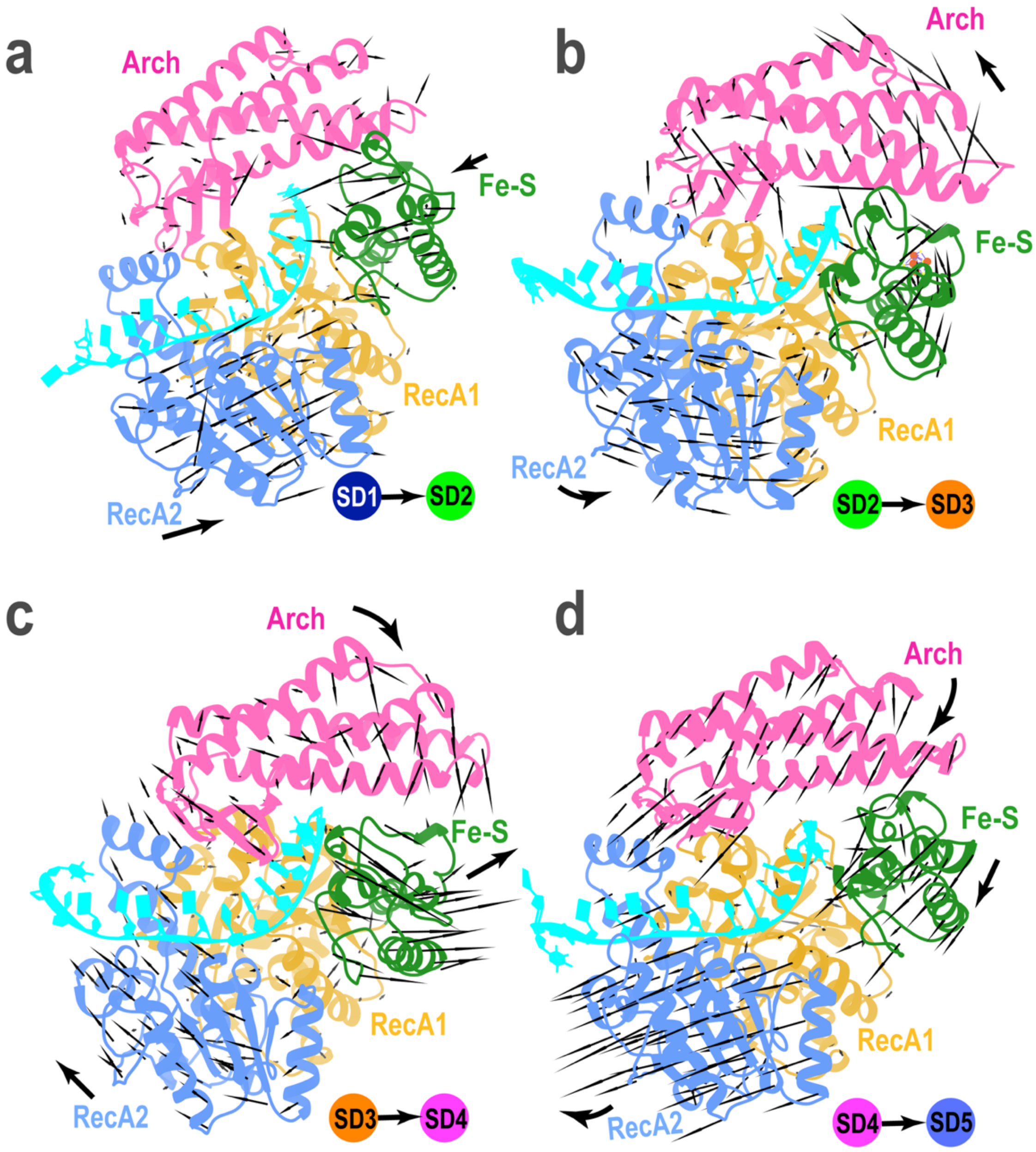

Supplementary Figure 13. Conformational switching of DinG's domains during the ATPase cycle. Porcupine plots showing the principal conformational changes in DinG during a single cycle of ATP binding and hydrolysis. DinG domains are colored as follows: RecA1 (gold), RecA2 (blue), Arch (magenta), and Fe–S (green). Gray arrows indicate the direction of Cα atom displacements during structural transitions among the five MSM macrostates (SD1-SD5): (**a**) SD1→SD2; (**b**) SD2→SD3; (**c**) SD3→SD4; (**d**) SD4→SD5. Black arrows indicate the overall direction of large-scale domain movements. Terminal conformational states for each transition are indicated in the bottom right corner of each panel.

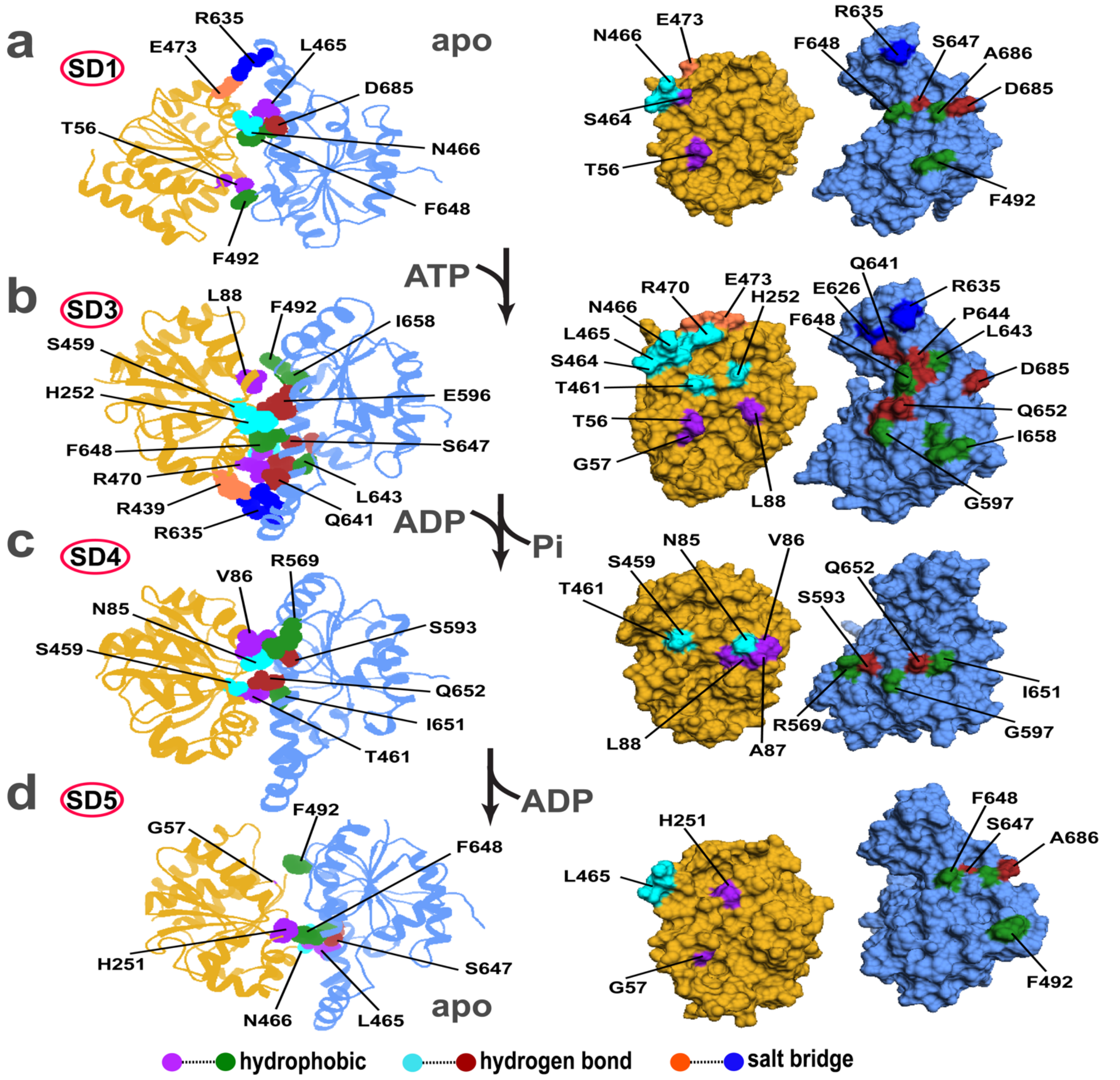

Supplementary Figure 14. **Changes in persistent residue contacts at the RecA1-RecA2 interface of DinG during the ATPase cycle.** A zoomed-in view of the DinG ATPase cleft in states: (**a**) SD1; (**b**) SD3; (**c**) SD4; and (**d**) SD5. Panels on the left show DinG residues that are key for the structural integrity of the motor domains interface. Panels on the right show the same key contacts mapped onto the surfaces of the RecA1 (gold) and RecA2 (blue) domains in 'open-book' representation. Color-coding is by interaction type: 1) salt bridge forming residues are in red and blue, 2) hydrogen bonding residues are in cyan and dark brown, and 3) residues involved in hydrophobic contacts are in purple and green.

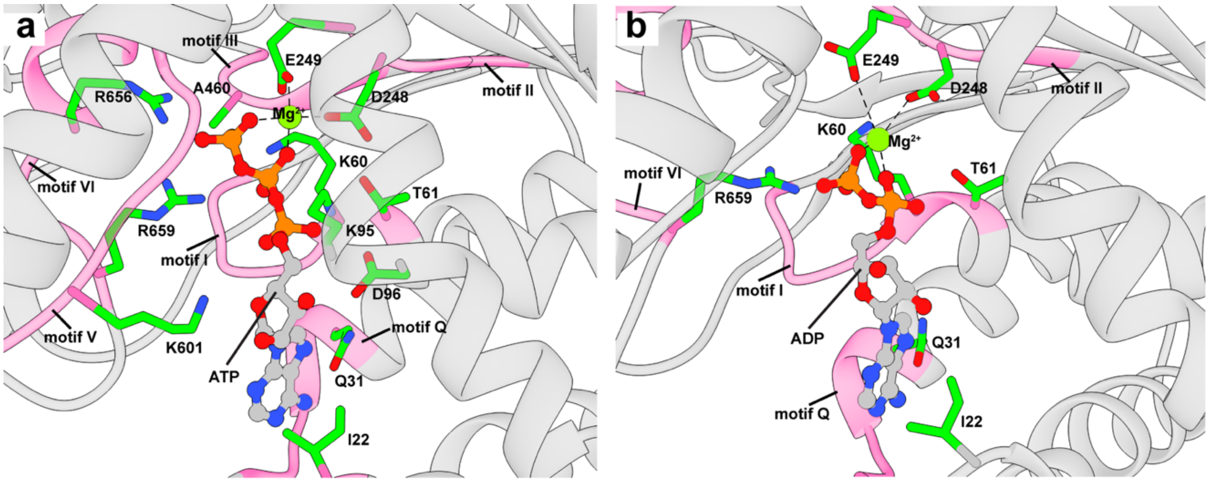

Supplementary Figure 15. Persistent residue contacts to the bound nucleotide in the DinG ATPase site. The DinG active site shown with bound nucleotide: (**a**) ATP and (**b**) ADP, respectively. The nucleotide is shown in ball-and-stick representation. Interacting residues are shown in stick representation and colored in green. The conserved helicase motifs involved in ATP and ADP binding are shown in pink and labeled.

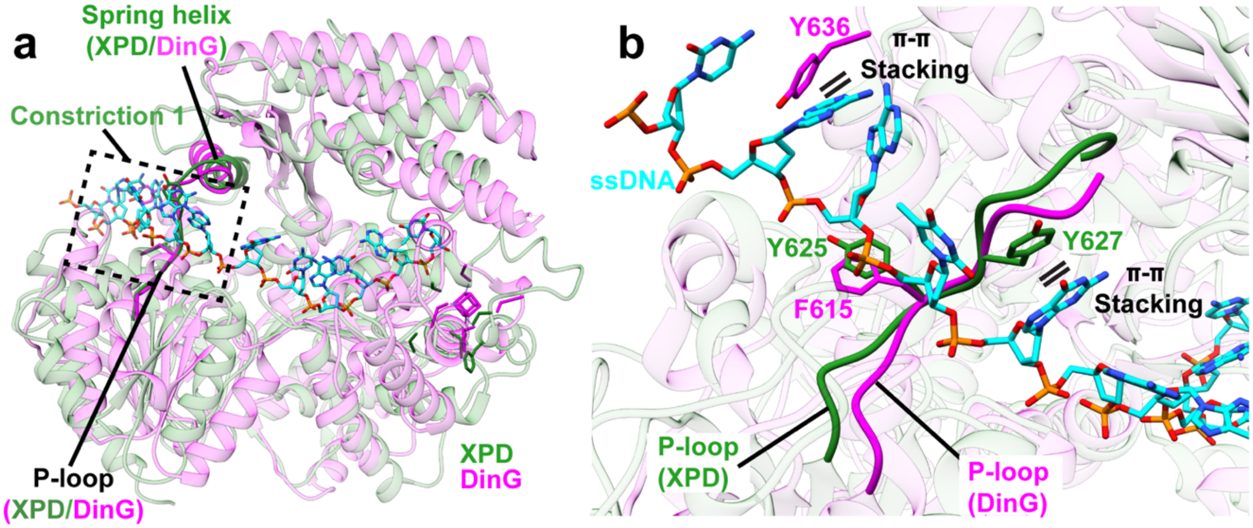

Supplementary Figure 16. Comparison of the P-loop conformations in XPD and DinG. a Positioning of the P-loop with respect to Constriction 1 (black dashed line rectangle) in XPD (green) and DinG (magenta). **b** Zoomed-in view of the P-loop in XPD and DinG showing key residues forming stacking interactions with ssDNA. Residues are shown in stick representation for XPD (green) and DinG (purple). The structural alignment is performed using the apo-XPD (PDB ID: 6RO4) and apo-DinG (PDB ID: 6FWR) structures.

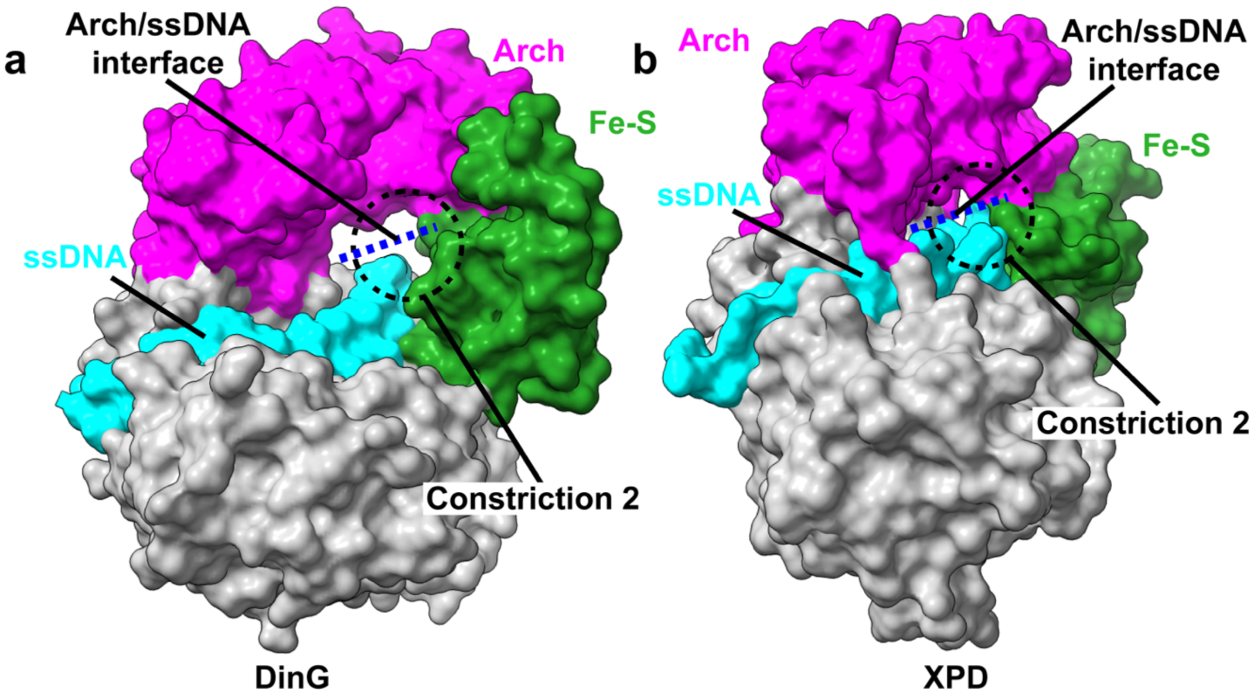

Supplementary Figure 17. **DNA accommodation within Constriction 2 in XPD and DinG in the apo state.** **a** DinG shown in surface representation with the position of Constriction 2 highlighted by a black dashed line circle. **b** XPD shown in surface representation with the position of Constriction 2 highlighted by a black dashed line circle. Domains are colored as follows: Arch (magenta), Fe–S (green), core motor domains (gray) and bound ssDNA (cyan). Interfaces between the Arch domain and ssDNA are shown by blue dashed lines. Surface representations are generated using the cryo-EM structure of XPD (PDB ID: 6RO4) and the crystal structure of DinG (PDB ID: 6FWR).

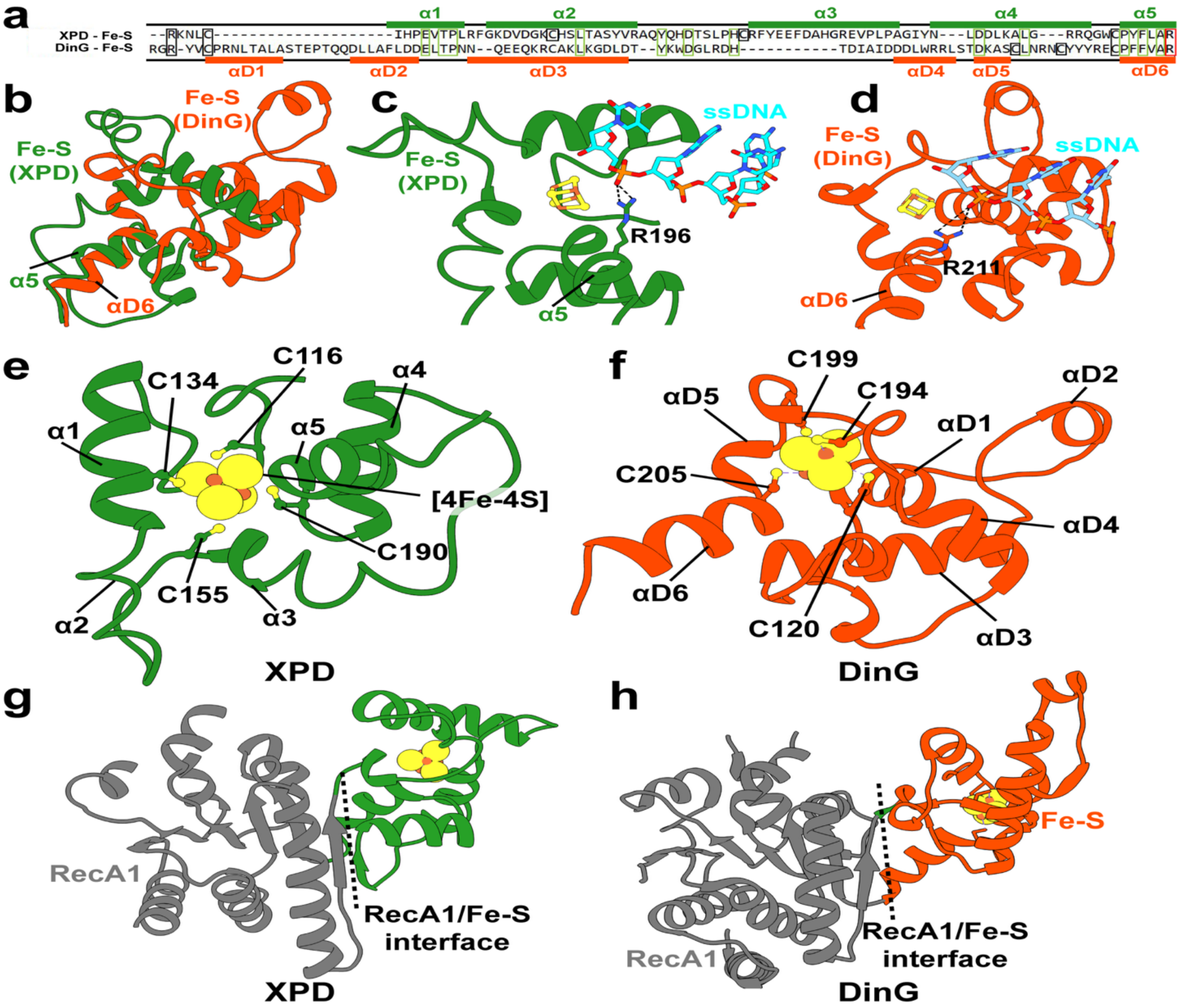

Supplementary Figure 18. Structural and sequence comparison of the Fe–S domains in XPD and DinG. a Sequence alignment of the XPD and DinG Fe–S domains; α-helices are represented as colored bars. Cysteine residues coordinating the iron–sulfur cluster outlined by black boxes. Conserved residues are outlined by green boxes and DNA-contacting residues by red boxes. **b** Superposition of the Fe–S domains of XPD (green) and DinG (red). Helices α6 in XPD and α7 in DinG are labeled. **c** Conserved arginine residue R196 of XPD interacting with ssDNA. **d** Analogous interaction between R211 of DinG and ssDNA. Positioning of the [4Fe-4S] binding cysteine residues for **e** XPD and **f** DinG. Cysteine residues are shown in ball stick representation and labelled. The iron-sulfur clusters of XPD and DinG are shown as spheres. **g** RecA1 – Fe–S interface of XPD. **h** RecA1 – Fe–S interface of DinG.

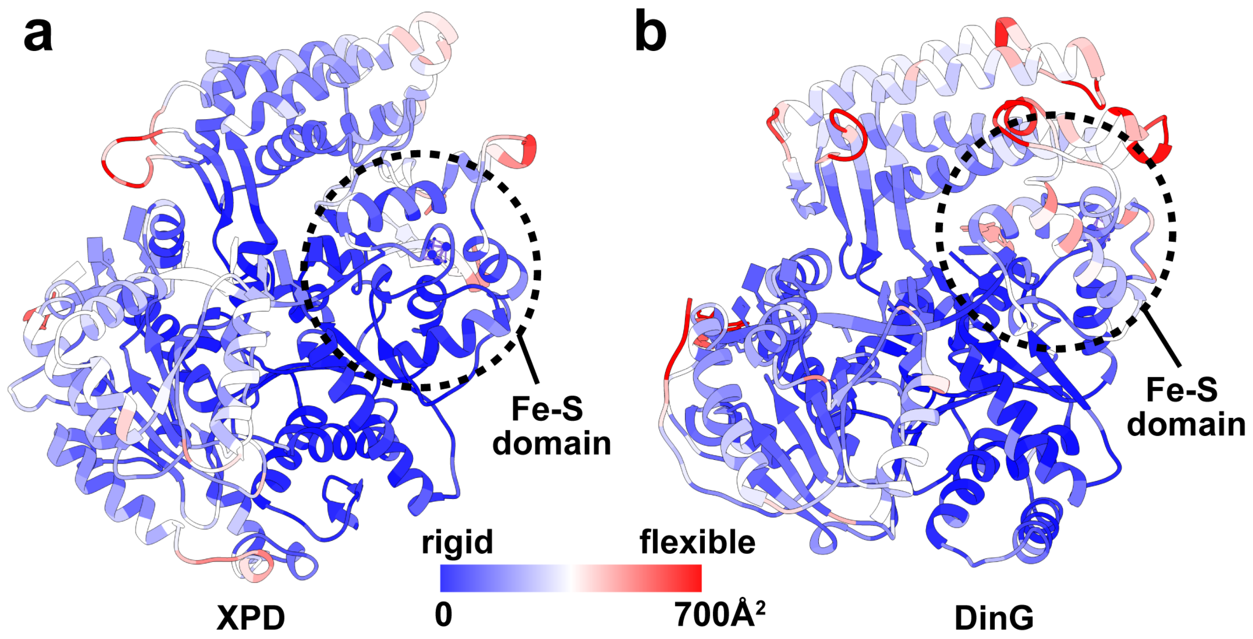

Supplementary Figure 19. Comparison of the relative flexibility of the Fe–S domains of XPD and DinG. B-factor values are mapped onto the structures of **a** apo-XPD and **b** apo-DinG, with rigid regions shown in blue and flexible regions in red. The Fe-S domains are highlighted with black dashed-line circles.

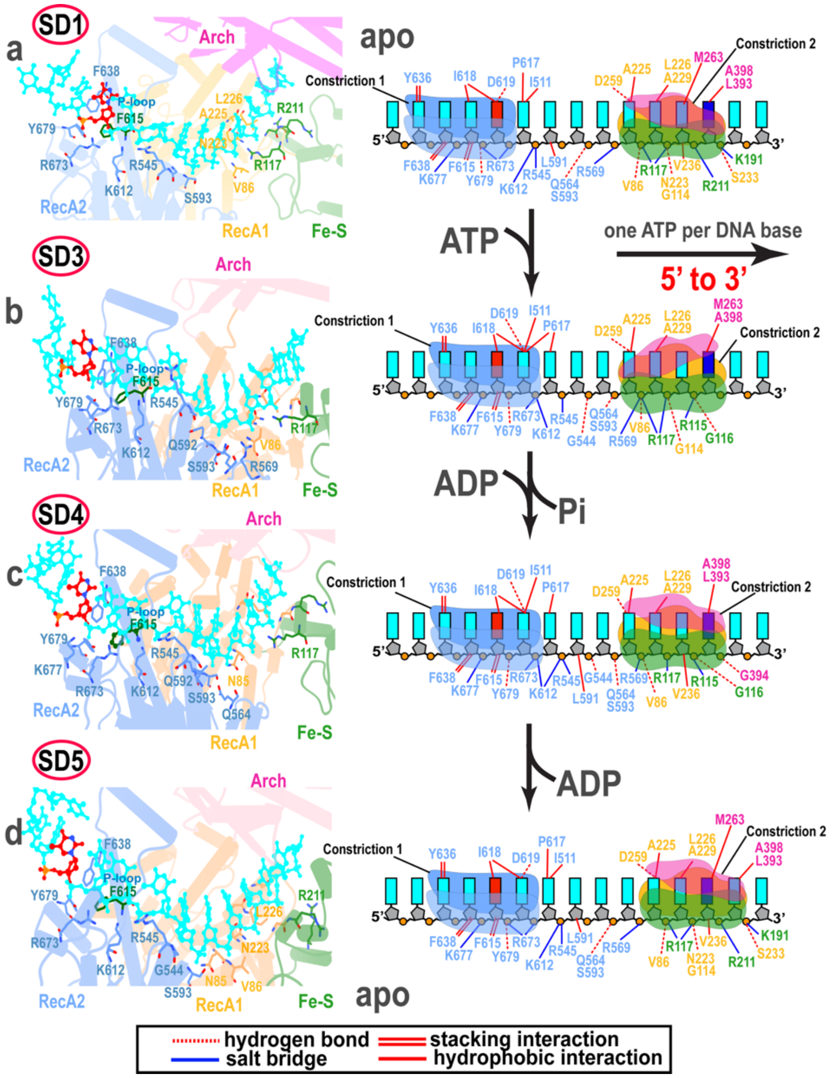

Supplementary Figure 20. Conformational switching of DinG's domains during the ATPase cycle causes dramatic changes in the ssDNA binding interactions. Zoomed-in views of the DNA-binding groove of DinG show the evolving contacts between XPD and ssDNA across four functional states: (**a**) SD1, (**b**) SD3, (**c**) SD4, and (**d**) SD5. Panels on the left show the DNA binding cleft with bound ssDNA (cyan) and DinG's domains colored as follows: RecA1 (gold), RecA2 (blue), Arch (magenta), and Fe–S (green). Key residue contacts to ssDNA are shown explicitly in atomic (ball-and-stick) representation. Side chains of interacting residues are colored by domain. Constriction1 and Constriction 2 and their engagement to ssDNA are shown schematically on the right-side panels, which also depict ssDNA interactions identified by persistent contact analysis of the four macrostates. Interaction types are denoted as follows: hydrogen bonds (red dashed lines), salt bridges (blue solid lines), base-stacking interactions (red parallel lines), and hydrophobic contacts (red solid lines).

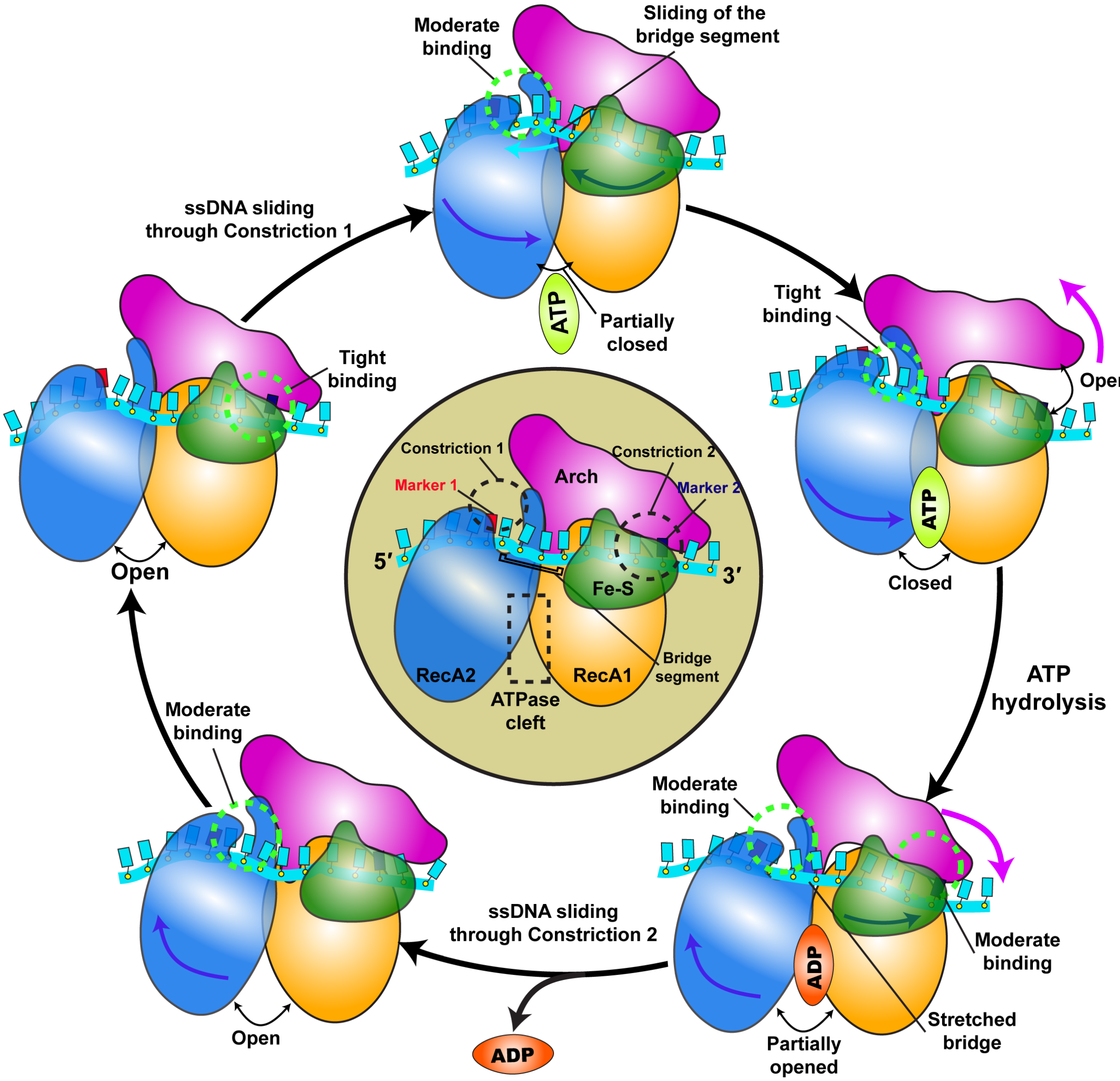

Supplementary Figure 21. Schematic of DinG translocation on ssDNA. DinG's four domains are color-coded: RecA1 (gold), RecA2 (blue), Arch (magenta), and Fe–S (green), and the ssDNA is shown in cyan. Key features—Constrictions 1 and Constrictions 2 (black dashed circle) and the ATPase cleft (black dashed rectangle) are highlighted. Marker 1 (red) and Marker 2 (navy) indicate the entry points to the Constriction 1 and Constrictions 2, respectively. Change in nucleotide states are labeled above arrows. For each step, the constriction engaged in tight ssDNA binding is marked with a light green dashed circle. Arrows indicate the directions of domain movements, matching the color of the moving domain.

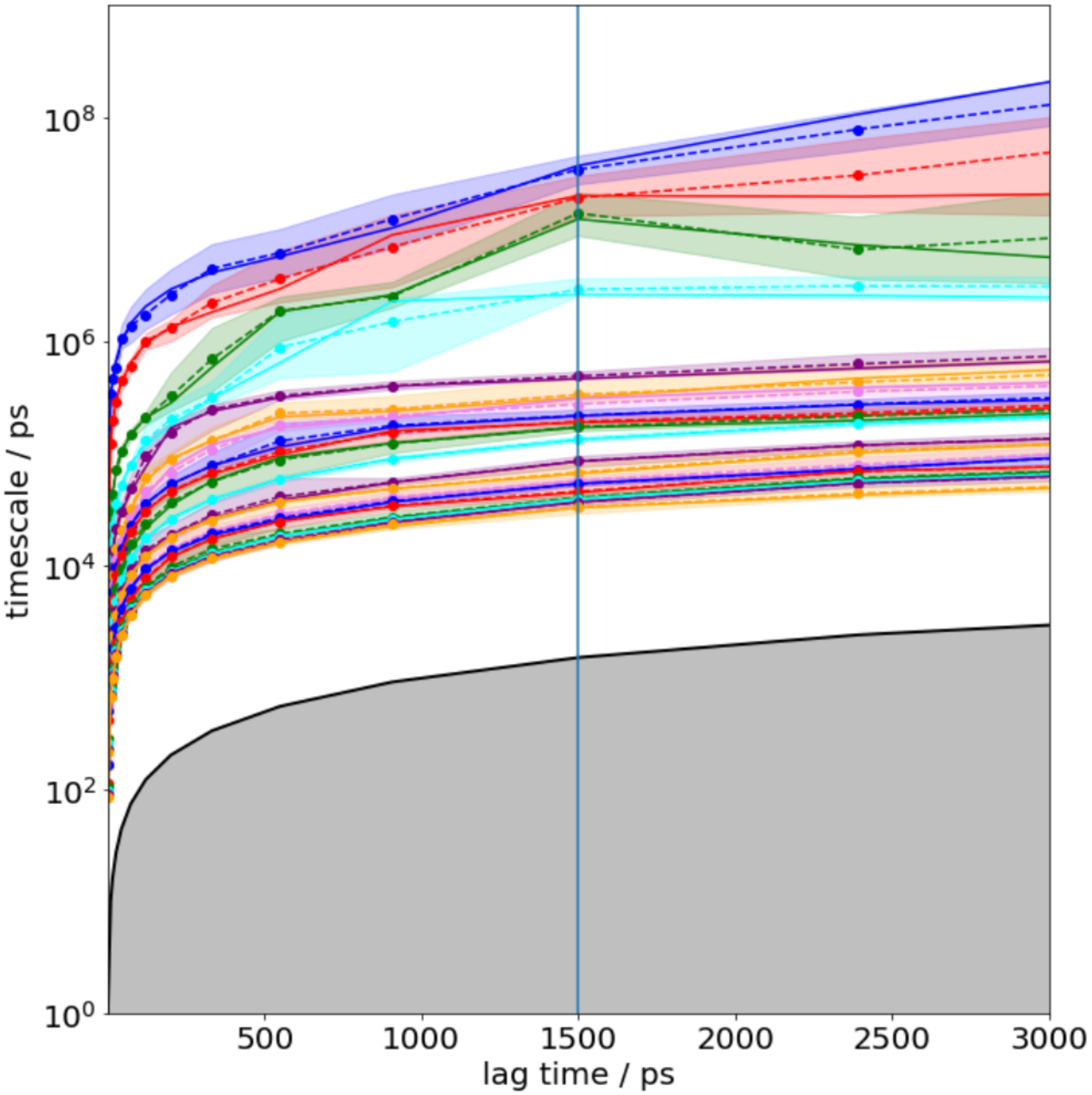

Supplementary Figure 22. Implied timescale plot showing the slowest processes that dominate the dynamics of XPD translocation. The ten slowest processes observed in the analysis of XPD dynamics are shown as colored lines. Shaded regions correspond to the 95% confidence intervals calculated using a bootstrap algorithm. These provide a measure of the uncertainty for each timescale. Source data are provided as a Source Data file.

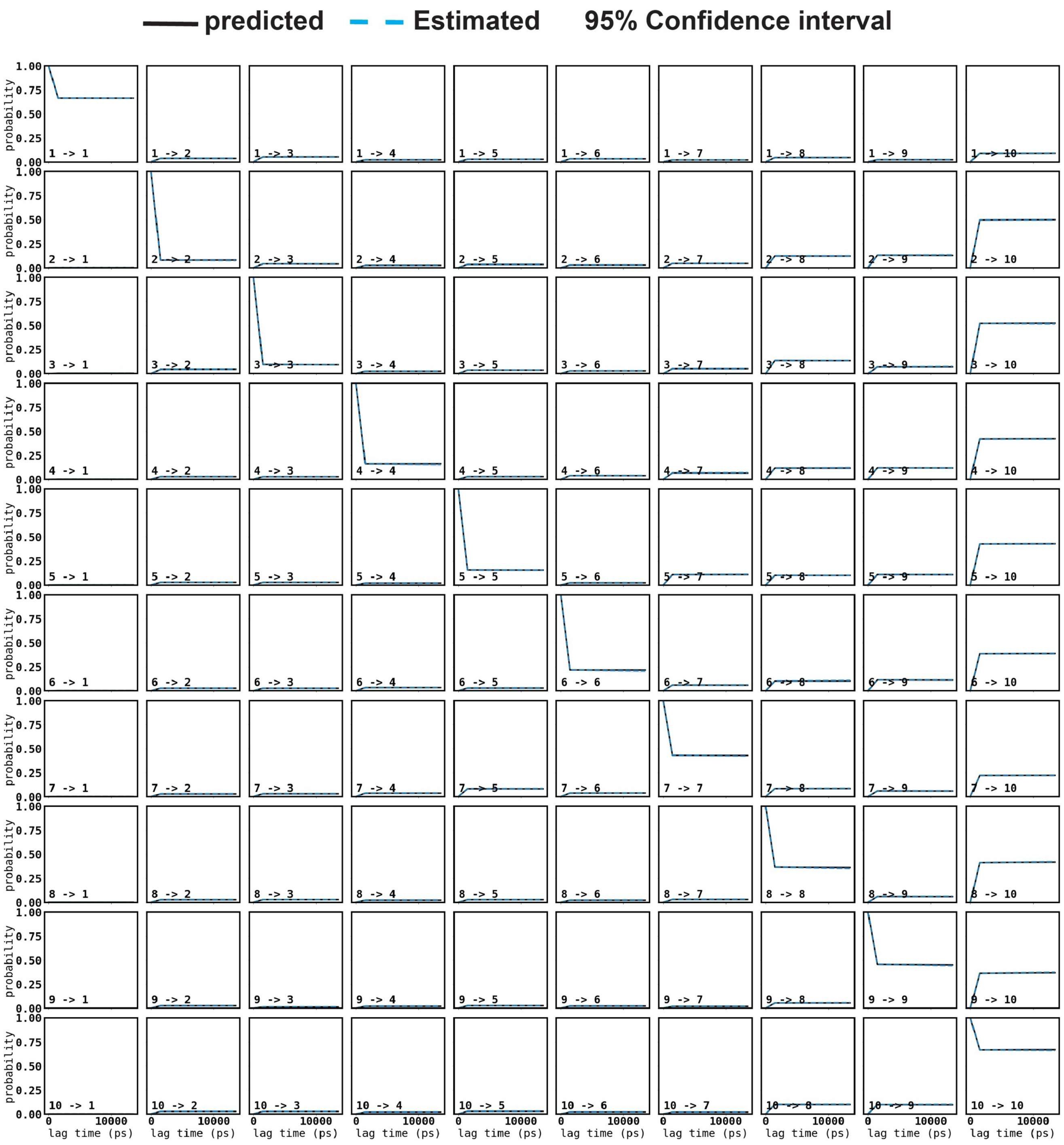

Supplementary Figure 23: **Validation of the Markov State Model for XPD using the Chapman-Kolmogorov test.** Comparison between the predicted (solid black line) and estimated data (blue dotted lines) are shown for the XPD MSM. This analysis confirms the accuracy of the MSM in describing XPD's conformational dynamics.
