# Supplementary material for: Translocation mechanism of xeroderma pigmentosum group D protein on single-stranded DNA and genetic disease etiology
Source: Nat Commun. 2025 Nov 28;16:11703. doi: 10.1038/s41467-025-66834-1 (PMC12753804; doi:10.1038/s41467-025-66834-1)
Supplement: Supplementary file 2 — Description of Additional Supplementary Files [file 41467_2025_66834_MOESM2_ESM.pdf]

Description of Additional Supplementary Files

File Name: Supplementary Data 1
Description: Initial and final frames from the MD trajectories

File Name: Supplementary Movie 1.
Description: Structural features of XPD underpinning the translocation mechanism. Domains are color-coded as shown in Fig. 1, with ssDNA in cyan. Marker 1 and Marker 2 are highlighted in red and dark blue, respectively. Key regions involved in ssDNA translocation, including Constriction 1, Constriction 2, the bridge segment, ATPase cleft, and conserved helicase motifs, are shown.

File Name: Supplementary Movie 2.
Description: Domain rearrangements in XPD shown over three consecutive ATPase cycles. XPD is colored by domain with ssDNA depicted in cyan. Marker 1 and Marker 2 on the ssDNA are highlighted in red and dark blue, respectively. Key residues interacting with the ssDNA at the two constrictions, Constriction 1 and Constriction 2, are highlighted.

File Name: Supplementary Movie 3.
Description: Mapping of XP, XP/CS, TTD, and XP/TTD mutations onto the XPD structure. Domains are colored as in Fig. 1, with disease mutations shown as spheres colored by phenotype. Class A, B, and C mutations are shown.

File Name: Supplementary Movie 4.
Description: Structural overview of DinG. Domains are color-coded as follows: RecA1 in gold, RecA2 in blue, Arch in magenta, and Fe–S in green. ssDNA is shown in cyan. Key regions involved in ssDNA movement such as Constriction 1, Constriction 2, and the ATPase cleft are shown.

File Name: Supplementary Movie 5.
Description: Domain rearrangements in DinG across three consecutive ATPase cycles. DinG is color-coded by domain as in Movie 4, with ssDNA shown in cyan. Marker 1 and Marker 2 on the ssDNA are highlighted in red and dark blue, respectively. Key residues at Constriction 1 and Constriction 2, interacting with the ssDNA, are highlighted.
